# Changes in DNA methylation are associated with the development of drug resistance in cervical cancer cells

**DOI:** 10.1186/s12935-015-0248-3

**Published:** 2015-10-13

**Authors:** Chih-Cheng Chen, Kuan-Der Lee, Mei-Yu Pai, Pei-Yi Chu, Chia-Chen Hsu, Chia-Chen Chiu, Li-Tzong Chen, Jang-Yang Chang, Shu-Huei Hsiao, Yu-Wei Leu

**Affiliations:** Department of Hematology and Oncology, Chang Gung Memorial Hospital, Chiayi, Chang Gung University College of Medicine, Taoyuan, Taiwan; Chang Gung Institute of Technology, Taoyuan, Taiwan; Department of Life Science, Human Epigenomics Center, Institute of Molecular Biology and Institute of Biomedical Science, National Chung Cheng University, Chiayi, 621 Taiwan; Department of Pathology, Show Chwan Memorial Hospital, Changhua City, Taiwan; National Institute of Cancer Research, National Health Research Institutes, Zhunan, Miaoli County 350 Taiwan; Division of Hematology/Oncology, Department of Internal Medicine, National Cheng Kung University Hospital, College of Medicine, National Cheng Kung University, Tainan, 704 Taiwan

**Keywords:** DNA methylation, DRUG, Cancer

## Abstract

**Background and propose:**

Changes in DNA methylation are associated with changes in somatic cell fate without the alteration of coding sequences. In addition to its use as a traceable biomarker, reversible DNA methylation could also serve as a therapeutic target. In particular, if the development of drug resistance is associated with changes in DNA methylation, then demethylation might reverse the resistance phenotype. The reversion of the drug-resistance might then be feasible if the association between abnormal DNA methylation and the development of drug-resistance could be identified.

**Methods:**

Methylation differences between the drug-resistance cervical cancer cell, SiHa, and its derived oxaliplatin-resistant S3 cells were detected by methylation specific microarray. The drug-resistance cells were treated with demethylation agent to see if the resistance phenotype were reversed. Targeted methylation of one of the identified locus in normal cell is expected to recapitulate the development of resistance and a two-component reporter system is adopted to monitor the increase of DNA methylation in live cells.

**Results:**

In this report, we identified methylation changes, both genome-wide and within individual loci, in the oxaliplatin-resistant cervical cancer cell S3 compared with its parental cell line SiHa. Treatment of S3 with a demethylation agent reversed increases in methylation and allowed the expression of methylation-silenced genes. Treatment with the demethylation agent also restored the sensitivity of S3 to cisplatin, taxol, and oxaliplatin to the same level as that of SiHa. Finally, we found that methylation of the target gene *Casp8AP2* is sufficient to increase drug resistance in different cells.

**Conclusions:**

These results suggest that global methylation is associated with the development of drug resistance and could serve as a biomarker and therapeutic target for drug resistance in cervical cancer.

**Electronic supplementary material:**

The online version of this article (doi:10.1186/s12935-015-0248-3) contains supplementary material, which is available to authorized users.

## Background

DNA methylation is a stable, dominant, and inheritable epigenetic modification that silences genes in somatic cells [1–3]. Through DNA methylation, environmental factors such as growth factors, food, and toxins can reshape the methylome and eventually differentiate or transform a cell [4, 5]. For example, knockdown of upstream estrogen receptors (ERs) increases methylation within ER target genes [6]. Also, various concentrations of diets given to pregnant mice can lead to production of the methylation source *S*-adenosylmethionine (SAM), altering methylation level at the promoter regions of fur color reporter genes and causing variegated fur color in the offspring [7–9]. Furthermore, environmental toxins such as endocrine disrupters can change methylation states through different signaling pathways [10, 11]. All of these examples demonstrate that the methylome is subject to further modifications.

Changes in the methylome are associated with cellular transformation [2, 12–14]. Dramatic methylome changes can be initiated early during the production of germ line cells and even before implantation [15, 16]. Particular changes of the methylome are associated with the specification of different cell lineages during development [17, 18]. Deviating from a normal state, abnormal global hypomethylation or hypermethylation of tumor suppressor genes can induce cancer as revealed by genetic studies [19, 20]. The accumulation of abnormal DNA methylation can be found after tumor formation, metastasis, and the development of drug resistance, although it’s not easy to form connections between particular changes in methylation and specific transformation events [21].

Several changes in DNA methylation may affect cellular sensitivity to drug treatment. For example, increased DNA methylation within *BRCA1* promoter in ovarian cancer patients correlate with better platinum-based chemotherapy [22]. By contrast, hypermethylation of *MLH1* is associated with increased cisplatin resistance in an ovarian cancer cell line [23]. Also, hypermethylated *DAPK* in colon and breast cancers correlates with drug resistance [24, 25]. DAPK works through the tumor necrosis factor-related apoptosis-inducing ligand (TRAIL). Hypermethylation within the *TRAIL* gene correlates with drug resistance in lung cancer [26], and the reversal of *TRAIL* methylation by methylation inhibitor treatment restores sensitivity to drug treatment [27–29]. These findings suggest that abnormal DNA methylation might affect cell death pathways and the development of drug resistance in cancer [30, 31].

Identifying the methylation changes related to drug resistance might provide a diagnostic clue as to whether the development of drug resistance is methylation-dependent. Also, if changes in DNA methylation are sufficient to cause drug resistance in cancer, then the reversal of these changes might restore the sensitivity of cancer cells to drug treatment. In this report, we characterized the SiHa cancer cell-derived oxaliplatin-resistant cervical cancer cell line S3 [32]. Treatment with a methylation inhibitor reversed drug resistance, indicating that the development of resistance is methylation-dependent [33]. Differential methylation hybridization (DMH) microarray was performed to detect methylation changes associated with the development of drug resistance [34, 35]. Previously, demethylation of these target loci restored the expression of the target genes and their sensitivities to different cancer drugs [36–39]. Finally, we applied a two-component system to monitor DNA methylation of the identified target gene [17, 40] *Casp8AP2* (NM_001137667) and found that increased methylation was associated with a drug-resistant phenotype. These findings suggest the possibility of identifying changes in methylation that are related to drug resistance in cancer.

## Methods

### Cell culture, isolation, and characterization

Human mesenchymal stem cells (MSCs) were isolated and cultured as described by Lee et al. [41], and cell expansion was as described by Hsiao et al. [17, 41]. MDA-MB-231, SiHa, and S3 cells were cultured with L-15, Minimum Essential Medium (MEM; Invitrogen), and MEM with 2 μg/ml oxaliplatin, respectively. For all cells, the medium was supplemented with 10 % fetal bovine serum (Invitrogen), 100 mg/ml penicillin/streptomycin (Invitrogen), and 2 mM l-glutamine (Invitrogen).

### 5-Aza-2′-deoxycytidine (5-Aza) treatment

Cells were treated with 5 μM 5-aza or an equal volume of DMSO as a control for 5 consecutive days.

### Cloning of the human *Casp8AP2* promoter

Primers for the human *Casp8AP2* promoter are listed in Additional file 1: Table S1. Human MSC genomic DNA was used as a polymerase chain reaction (PCR) template. Purified PCR products were ligated into the *pyT&A* cloning vector (Yeastern Biotech) according to the manufacturer’s protocol. Inserts were confirmed by restrictions and sequencing.

### In vitro DNA methylation

PCR-amplified *Casp8AP2* promoters (4 μg) were incubated with 20 units of CpG methyltransferase (New England BioLabs) at 37 °C for 4 h in the presence of 160 μM SAM to induce methylation.

### Validation of in vitro DNA methylation

Methylated DNA showing resistance to methylation-sensitive restriction enzymes (*Hpa*II) was considered to indicate completed conversion (Additional file 1: Figure S1).

### Transfection of methylated DNA

PCR products (0.4 μg/well, unmethylated as a control) were denatured at 95 °C and then transfected into 5 × 10^5^ cells/well in a 6-well plate using DMRIE-C (Invitrogen) according to the manufacturer’s instructions. Cells were transfected three times, on day 1, 3, and 5 [17, 42]. The transfection efficiency and the localization of the transfected DNA were tracked as in Additional file 1: Figure S2.

### Bisulfite conversion

Genomic DNA (0.5 μg) was bisulfite-converted and purified as described by Yan et al. [43].

### Semi-quantitative real-time methylation-specific PCR (qMSP)

The qMSP was performed as described by Yan et al. [43]. Bisulfite-converted genomic DNA was subject to real-time PCR with methylation-specific primers (Additional file 1: Table S1). A SYBR Green I PCR Kit (Toyobo) was used to conduct qMSP in an iQ5 PCR instrument (Bio-Rad). After reactions, analysis of melting temperature was performed to ensure that a specific amplicon was generated. *Col2A1* (NM_033150) was used for standard curve construction and as a loading control. Methylation percentage was calculated as: (mean of target gene)/(mean of *Col2A1*). Fold change was calculated as: (targeted DNA methylation percentage)/(mock methylation percentage).

### Differential methylation hybridization microarray, DMH

The DMH procedure was performed as described by Leu et al. [44] using a human CpG microarray (Agilent). Treated and control genomic DNA (2 μg) was restricted into small fragments by *Mse*I and ligated with designated primers. Methylation-sensitive restriction enzymes (*Bst*UI and *Hpa*II) were used to discriminate between methylated and unmethylated DNAs, and DNAs was then amplified by PCR using adaptors as primers. PCR-amplified DNA from mock-treated S3 cells was labeled with Cy5 and from SiHa or 5-aza-treated S3 cells was labeled with Cy3 and then co-hybridized onto slides. After scanning, the ratio between Cy5 and Cy3 dyes was normalized by locally weighted scatterplot smoothing. Significant methylation differences were identified.

### Semi-quantitative RT-PCR (qRT-PCR)

RNA isolation, first-strand cDNA synthesis, and detection of transcripts were carried out as previously described [44]. Total RNA (2 μg) was reverse transcribed using SuperScript II reverse transcriptase (Invitrogen). qRT-PCR was performed using a SYBR Green I PCR Kit (Toyobo) in an iQ5 Real-Time instrument (Bio-Rad). A serial dilution of *GADPH*-amplified (NM_002046) cDNA was used as a control to generate a standard curve, and *GAPDH* from each sample was used as a loading control. The primers used are listed in Additional file 1: Table S1.

### Cell survival assay

Cells (5 × 10^4^) were plated into each well of a 96-well assay plate and allowed to attach. Cells were then treated with different concentrations of drugs and incubated at 37 °C overnight. 3-(4,5-Dimethylthiazol-2-yl)-2,5-diphenyltetrazolium bromide (MTT) solution (20 μl, 5 mg/ml; Sigma) was added to each well and incubated at 37 °C for 5 h. The reaction was terminated by adding 100 μl DMSO, and absorbance was measured at 595 nm.

### Western blot analysis

Cells were harvested in RIPA buffer, and proteins were separated in 10 % polyacrylamide gel and trans-blotted onto a membrane. After blocking with skim milk, the membrane was hybridized with designated antibodies. After washing, secondary antibody conjugated with horseradish peroxidase was used to detect hybridization. Results were visualized by chemiluminescence. The film was then scanned and analyzed.

### Immunostaining

Treated or control cells (5 × 10^4^) were plated into 4-well chamber slides and allowed to attach. After washing and fixing in 2 % formaldehyde, cells were again washed and permeabilized by 0.5 % NP40 in phosphate-buffered saline (PBS). After another wash, horse serum in PBS (1:100) was used to block, and the slides were washed. Specific antibodies were used to stain the cells, and fluorescein-conjugated secondary antibodies were used to detect the staining. The slides were mounted, and cells were visualized using a fluorescent microscope.

### Enzyme-linked immunosorbent assay (ELISA)

Green fluorescent protein (GFP) ELISA was performed using an ELISA Kit (Cell Biolabs) according to the manufacturer’s instructions. Starting from 2 × 10^4^ cells per assay, the cells were harvested and lysed, and the collected proteins were quantified. Proteins (0.1 μg/ml per assay) were compared with the provided standard after binding to GFP antibody, secondary antibody, and substrate solution with vigorous washes between steps. After stopping the reaction, absorbance was measured at 595 nm.

## Results

### Changes in DNA methylation and expression in oxaliplatin-resistant cervical cancer S3 cells

**Fig. 1:**
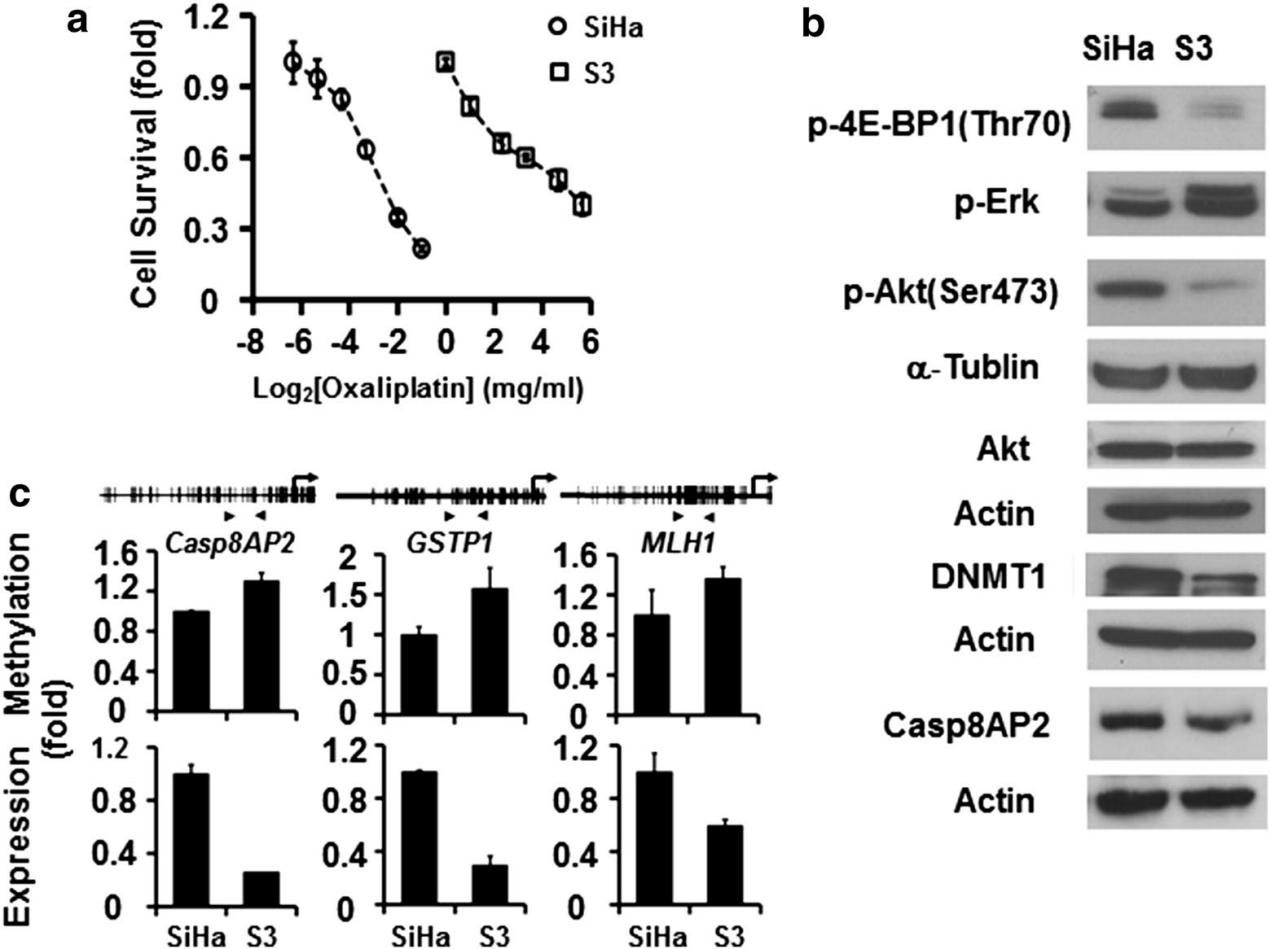
Gene methylation and expression changes in S3 cells. **a** Survival of S3 and SiHa cells upon treatment with different concentrations of oxaliplatin as assessed by MTT assay. **b** Differences in gene expression between S3 and SiHa cells as revealed by protein levels in western blot analysis. **c** Differences in gene methylation and expression between S3 and SiHa cells. Methylation within the promoter regions (*arrowheads*) was measured by qMSP (*upper panels*). Corresponding changes in gene expression were detected by qRT-PCR (*lower panel*)

A more than 40-fold decline in sensitivity was found for the oxaliplatin-resistant cervical cancer cell S3 compared with its parental SiHa cervical cancer cell line (Fig. 1a). The expression of several death-related genes decreased during the development of oxaliplatin resistance (Fig. 1b). This decreased expression of death-related genes, such as *Casp8AP2*, the detoxification gene *GSTp1*, and the repair gene *MLH1*, was associated with increased methylation within their promoter regions (Fig. 1c). These findings suggest that changes in epigenetic and DNA expression states are involved in the development of drug resistance.

### Global methylation changes in S3 cells

**Fig. 2:**
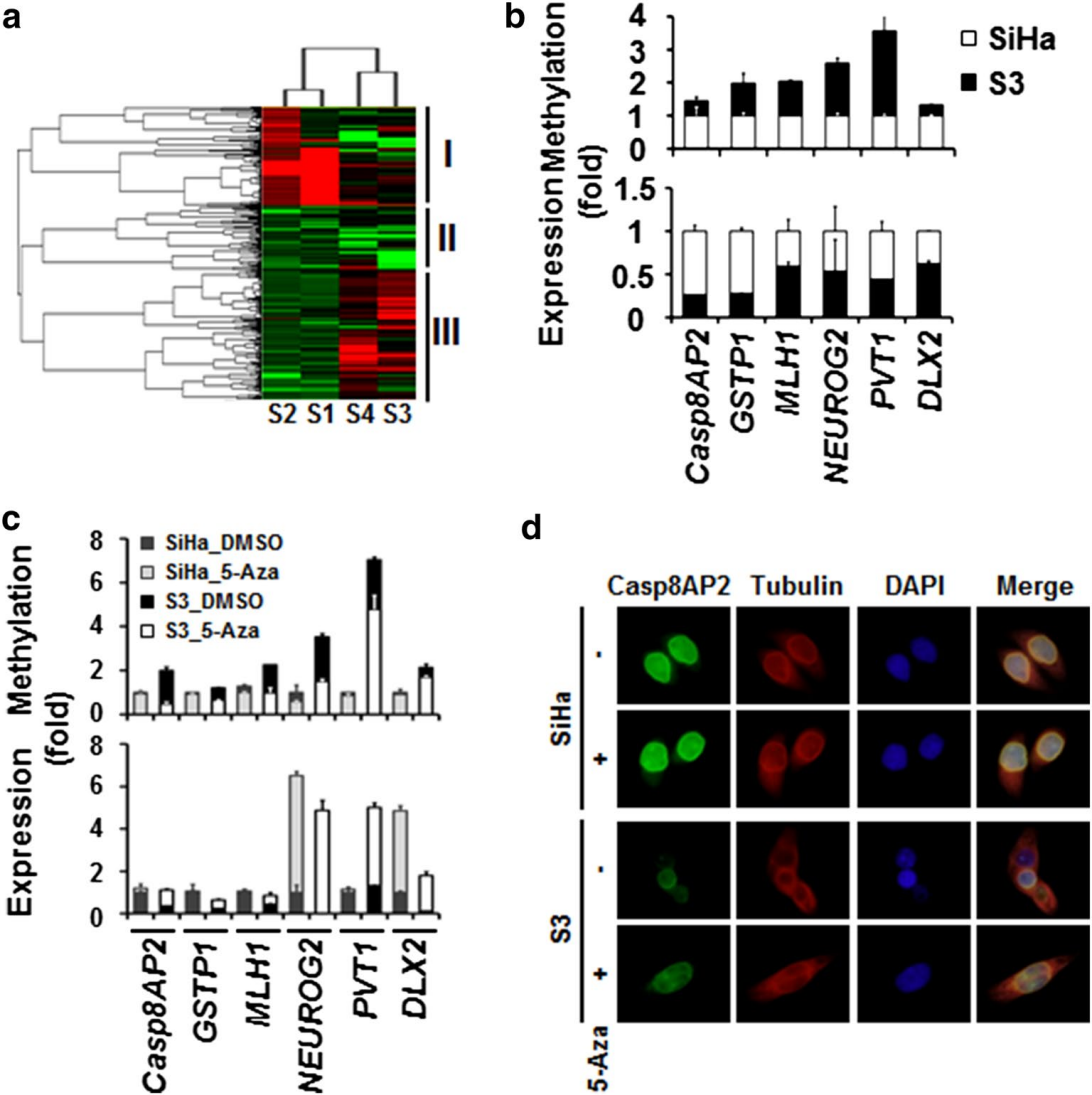
Global methylation changes in S3 cells. **a** DMH microarray detected differences in genome-wide methylation between S3 and SiHa cells (S1 and S2) and between 5-aza-treated and -untreated S3 cells (S3 and S4). These four sets of data were then subject to unsupervised hierarchical clustering analysis. Blocks I, II, and III designate the main clustering blocks. **b** Validation of gene methylation and expression differences between S3 and SiHa cells. Three genes (*NEUROG2*, *PVT1,* and *DLX2*) with identified methylation differences from **a** were validated by qMSP, and changes in their expression were detected by qRT-PCR. Three genes (*Casp8AP2*, *GSTP1,* and *MLH1*) with known methylation and expression differences between S3 and SiHa cells were used as controls. **c** These loci were hypermethylated in S3 cells, and their methylation was reversed (*upper panel*) and their expression restored (*lower panel*) by treatment with 5-aza. **d** Immunostaining confirmed the restoration of Casp8AP2 expression after treatment with 5-aza

Genome-wide changes in methylation during the development of drug resistance in S3 cells were detected by DMH. Changes in methylation were compared between S3 and SiHa cells (Fig. 2a, S1 and S2) as well as before and after 5-aza treatment in S3 cells (Fig. 2a, S3 and S4). After hierarchical clustering analysis of repeated DMH results, we found both increases (Fig. 2a, block I) and decreases (Fig. 2a, block III) in methylation within the S3 genome. Some of the increases in methylation were inhibited by 5-aza treatment (block I), suggesting that these methylation changes are associated with the development of drug resistance. On the other hand, some increases in DNA methylation were observed after 5-aza treatment (block III), suggesting that these methylation changes are not involved in the development of drug resistance. Three primary target loci (*NEUROG2*, *PVT1,* and *DLX2*) identified from DMH analysis were validated together with the three known targets (*Casp8AP2*, *GSTP1,* and *MLH1*) as controls (Fig. 2b). S3 showed increased methylation within the promoter region and lower expression of these genes compared with SiHa. This increased methylation (Fig. 2c, upper panel) and lower gene expression (Fig. 2c, lower) in S3 cells was reversed by 5-aza treatment. Treatment with 5-aza also increased Casp8AP2 expression in S3 cells as detected by RT-PCR (Fig. 2c) and immunostaining (Fig. 2d, also detected by Western blot, Additional file 1: Figure S3).

### Demethylation and reversal of drug resistance in S3

**Fig. 3:**
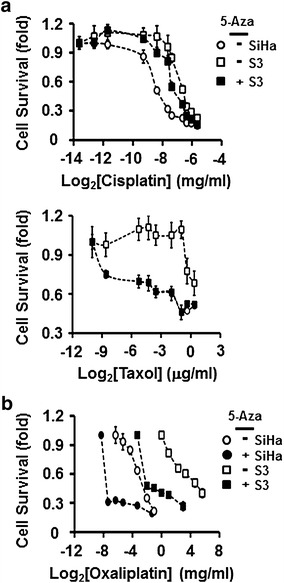
5-Aza treatment reverses drug resistance in S3 cells. **a** After S3 cells were mock-treated or treated with 5-aza, they were treated with different concentrations of cisplatin (*upper panel*) or taxol (*lower panel*). Mock-treated SiHa cells were used as a control. Cell survival was measured by MTT assay. **b** S3 and SiHa cells were treated with 5-aza to demethylate their DNA, and cells were then challenged with different concentrations of oxaliplatin. Cell survival was measured by MTT assay. The survival rates of untreated S3 and SiHa cells were included from Fig. 1 for comparison

If increased DNA methylation in drug-resistant cancer cells is necessary for the maintenance of drug resistance, then the reversal of DNA methylation in S3 should restore their sensitivity to drug treatment. After 5-aza treatment, S3 lost their resistance to cisplatin and taxol (Fig. 3a, upper and lower panels, respectively). Also, after 5-aza treatment, the sensitivity of S3 cells to oxaliplatin was restored to the same level as that in untreated SiHa (Fig. 3b). Together, these findings indicate that the maintenance of methylation may be critical for the maintenance of drug resistance in cancer cells.

### Visualization of the targeted *Casp8AP2* methylation and increased drug resistance in cells

**Fig. 4:**
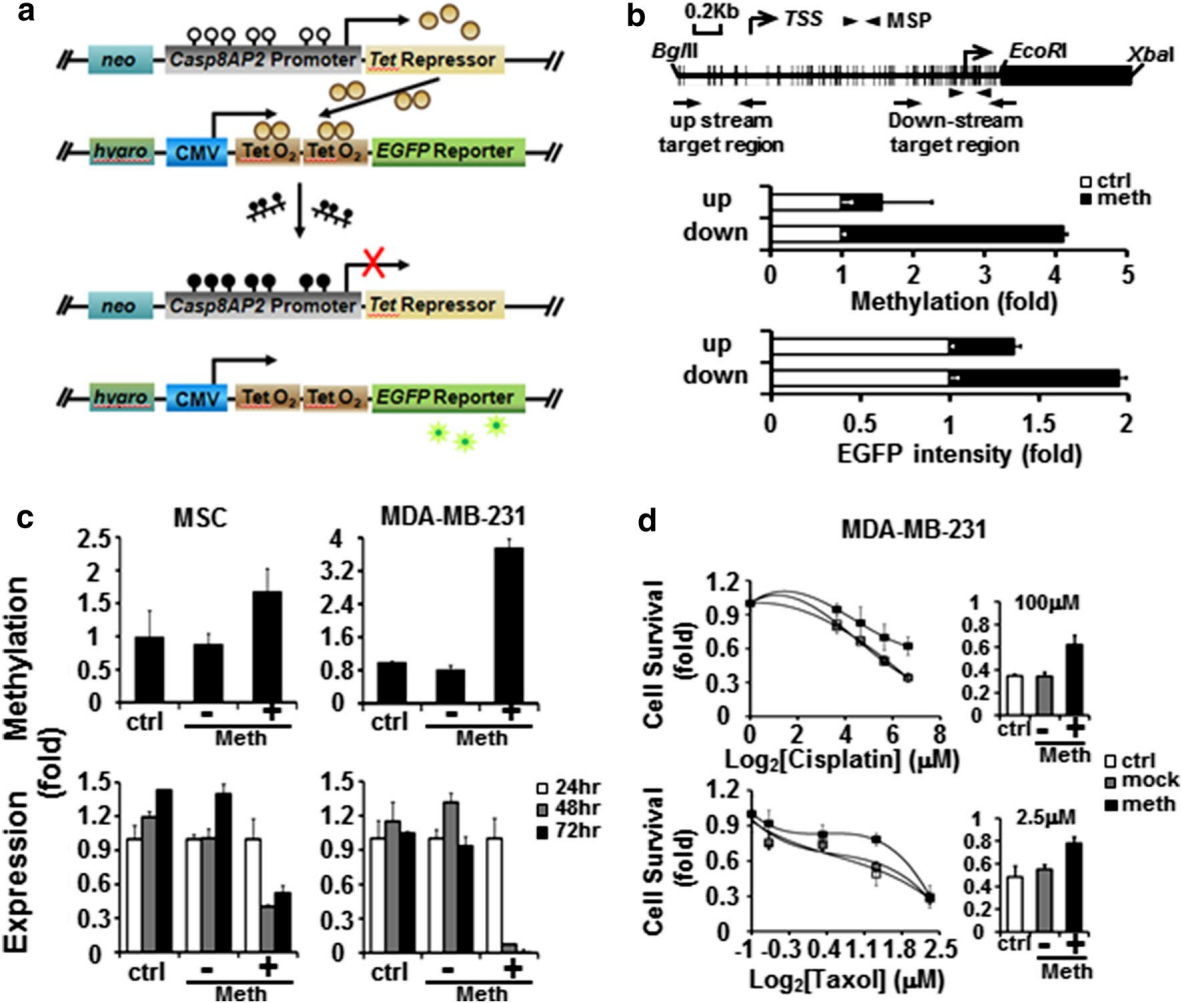
Targeted *Casp8AP2* methylation increases drug resistance. **a** Two-component system for visualizing targeted DNA methylation. Components were co-transfected into the same cells, and stable clones were selected by adding neomycin and hygromycin to the culture medium. Detection of targeted DNA methylation is described in the “Methods”. **b** Detection of targeted DNA methylation. *Upstream* and *downstream* target regions are indicated by the regions between two *arrows* in the *upper panel*. *Short vertical bars* indicate CpG sites. Targeted DNA methylation was detected by qMSP with primer pairs (indicated by *arrowheads* in the *upper panel*), and mock-treated cells were used as a control (*middle panel*). Expression of the *EGFP* reporter gene was detected by ELISA (*lower panel*). **c** Increased *Casp8AP2* methylation (*upper panel*) and decreased Casp8AP2 expression (*lower panel*) after targeted DNA methylation in MSCs (*left*) and MDA-MB-231 breast cancer cells (*right*). Untreated cells are designated as control (ctrl), and cells treated with transfection reagent only are designated as (−). **d** Increased cell survival after targeted *Casp8AP2* methylation. MDA-MB-231 cells were transfected with in vitro methylated (*S.ss*I) or unmethylated *Casp8AP2* DNA and then challenged with different concentrations of cisplatin (*upper panel*) and taxol (*lower panel*). Cell survival after drug treatment was detected by MTT assay

To confirm that methylation of specific genes is sufficient to increase cellular drug resistance, we developed a two-component system to monitor methylated *Casp8AP2*. To visualize the targeted methylation of *Casp8AP2*, the *Casp8AP2* promoter was cloned in front of the *Tet* repressor gene, and, on another vecor, Tet repressor binding sites *TetO2* (*Tet* operator) were cloned in front of the enhanced *GFP* (*EGFP*) reporter (Fig. 4a). If *Casp8AP2* is not methylated, the Tet repressor is expressed, binds to *TetO2* and suppresses *EGFP* expression. By contrast, the targeted methylation of *Casp8AP2* suppresses *Tet* repressor gene expression and releases the suppression of *EGFP* expression. Using this system, *Casp8AP2* methylation is reflected by increased levels of EGFP.

We chose both an upstream and downstream regions to targeted methylate *Casp8AP2* (Fig. 4b, upper panel). We observed increases in *Casp8AP2* methylation (Fig. 4b, center panel) and EGFP expression (Fig. 4b, lower panels) regardless of whether the far or near ends were targeted. The targeted *CAsp8AP2* methylation was also evidenced by bisulfite sequencing (Additional file 1: Figure S4) Successful targeted DNA methylation increased the methylation (Fig. 4c, upper panels) and reduced gene expression (Fig. 4c, lower panels) of *Casp8AP2* in both MSCs and the breast cancer cell line MDA-MB-231. Both cell lines became less sensitive to cisplatin and taxol after *Casp8AP2* methylation (MDA-MB-231 cells: Fig. 4d; MSCs: Additional file 1: Figure S5).

## Discussion

DNA methylation is an inheritable mark that could direct gene expression and cell fates [45, 46]. Changes in DNA methylation often imply a detour in cell physiology and could serve as a way to further vary cellular transformation and clonal expansion [47, 48]. Accumulating data correlates abnormal DNA methylation with tumorigenesis, metastasis, and the development of drug resistance [47, 49]. Although genetic studies directly link abnormal DNA methylation to cellular transformation, how abnormal DNA methylation leads to the development of drug resistance is relatively unclear.

DNA methylation is a stable change, yet it is also reversible like other epigenetic modifications. Its stability makes methylation easy to detect, and its reversibility makes it a possible therapeutic target [50, 51]. If methylation of a specific locus is sufficient to cause drug resistance, then detection of this modification might be used to monitor the development of drug resistance. Furthermore, if demethylation of a locus is closely related to the reversal of drug resistance, then it could be a candidate mechanism for restoring the sensitivity of cells to drug treatment. Therefore, we used DMH to identify several methylation changes occurring during the development of drug resistance. The loci that became hypermethylated and that showed a reversal of methylation after treatment with a demethylation agent could be primary targets [52].

In the present study, by establishing a two-component system for monitoring targeted DNA methylation and quantifying the degree of methylation, we found that targeted *Casp8AP2* methylation caused the development of cellular drug resistance in different lines of cells. This monitoring system could be further used to monitor environmentally induced changes in methylation state and to track targeted cells in their microenvironments.

## Conclusion

Global methylation changes are associated with the development of drug resistance in cervical cancer. Methylation inhibitors reverse the resistance-associated methylation increases and the resistance phenotype. Methylation changes might serve as a biomarker and therapeutic target for drug resistance in cervical carcinoma cells.

## Additional file

10.1186/s12935-015-0248-3 DNA methylation change is associated with the development of drug resistance in cervical cancer.

## Abbreviations

5-Aza: 5-aza-2′-deoxycytidine
SAM: *s*-adenosylmethionine
MSC: mesenchymal stem cell
