# Supplementary material for: Changes in DNA methylation are associated with the development of drug resistance in cervical cancer cells
Source: Cancer Cell Int. 2015 Oct 13;15:98. doi: 10.1186/s12935-015-0248-3 (PMC4604021; doi:10.1186/s12935-015-0248-3)
Supplement: Supplementary file 1 — 10.1186/s12935-015-0248-3 DNA methylation change is associated with the development of drug resistance in cervical cancer. [file 12935_2015_248_MOESM1_ESM.docx]

**DNA methylation change is associated with the development of drug resistance in cervical cancer**

Chih-Cheng Chen, Kuan-Der Lee, Mei-Yu Pai, Pei-Yi Chu, Chia-Chen Hsu, Chia-Chen Chiu, Li-Tzong Chen, Jang-Yang Chang, Shu-Huei Hsiao and Yu-Wei Leu

Table S1. Primer used.

Figure S1. Restriction validation of *in vitro Casp8AP2* methylation.

Figure S2. Transfection and localization of the transfected DNAs.

Figure S3. Restored Casp8AP2 expression after 5-Aza treatment.

Figure S4. Bisulfite sequence analysis of the increased DNA methylation after

targeted DNA methylation.

Figure S5. Increased MSC survival after targeted *Casp8AP2* methylation.

Table S1. Primer used.

| Primer name | Gene RefSeq | Sequence | Detection |
| --- | --- | --- | --- |
| H_GAPDH_RT_F | *GAPDH* NM_002046 | CCCCTTCATTGACCTCAACTAGAT | RT PCR Control |
| H_GAPDH_RT_R |  | CGCTCCTGGAAGATGGTGA |  |
| BR_137 | *Col2A1*  NM_0033150 | TCTAACAATTATAAACTCCAACCACCAA | MSP Control |
| BR_138 |  | GGGAAGATGGGATAGAAGGGAATAT |  |
| H_GSTP1_RT_F | *GSTP1*  NM_000852 | GGGCAGTGCCTTCACATAGT | RT PCR |
| H_GSTP1_RT_R |  | GGAGACCTCACCCTGTACCA |  |
| H_GSTP1_F |  | AAGGTTAGGAGTTCGAGATTAGTTC | MSP |
| H_GSTP1_R |  | CCCGAATAAATAAAATTATAAATACGT |  |
| H_Casp8AP2_1A_F | *Casp8AP2*  NM_012115.3 | GTGAAGGTAATCATCCTGCATTA | RT PCR |
| H_Casp8AP2_1A_R |  | GAACTGGGAGATTCTGTGGT |  |
| H_Casp8AP2_promoter_F |  | AATAAAATAGTTAGGGGTGGTGGTC | MSP |
| H_Casp8AP2_promoter_R |  | TTCAAACAAAATATCGCTTTATCGC |  |
| H_Casp8AP2_TSS_F |  | GATTATTGGAGTTTGGCGTTAATC |  |
| H_Casp8AP2_TSS_R |  | CGAAACTAAATACCTACGACCAATCATA |  |
| H_Casp8AP2_exon_F |  | GTTGTTTTTGGGAAATTAGAGAGTC |  |
| H_Casp8AP2_exon_R |  | AACGAAAAAAACGTAACTACCTAC |  |
| H_Casp8AP2_pro_insertion_F |  | GGTGGTGGTCGCTCGCGTCTA | Cloning |
| H_Casp8AP2_pro_insertion_R |  | CCTCATTAAGCAGCTCTAATGCGCTG |  |
| HGMP1.1 |  | CGGCCGCTGCAGGTCTGACCATAA | CpG clone |
| HGMP2.1 |  | AACGCGTTGGGAGCTCTCCCATAA |  |
| H_MLH1_RT_F1 | *MLH1*  NM_000249 | GAAAACTGAAAGCCCCTCCT | RT PCR |
| H_MLH1_RT_R1 |  | ACGGTTGAGGCATTGGGTAGT |  |
| H_MLH1_MSP_F1 |  | ACGTAGACGTTTTATTAGGGTCGC | MSP |
| H_MLH1_MSP_R1 |  | CC TCATCGTAACTACCCGCG |  |

Table S1 (continued). Primer used for target gene validation

| Primer name | Gene RefSeq | Sequence | Detection |
| --- | --- | --- | --- |
| H_NEUROG2_RT_F | *NEUROG2*  NM_024019 | CGCATCAAGAAGACCCGTAGA | RT PCR |
| H_ NEUROG2_RT_R |  | GTGAGTGCCCAGATGTAGTTGT |  |
| H_NEUROG2_MSP_F |  | ACGGATTTTAAATATATTTGTTTACGA | MSP |
| H_NEUROG2_MSP_F |  | TAACCCTATAATACTACCGAACGTC |  |
| H_PVT1_RT_F | *PVT1*  NR_003367 | GGAGGCTGAGGAGTTCACTGA | RT PCR |
| H_ PVT1_RT_R |  | TTGGGGCAGAGATGAAATCGTAAT |  |
| H_ PVT1_MSP_F2 |  | TTTAGTAGGAAAGTGGGAAGATCGT | MSP |
| H_ PVT1_MSP_R2 |  | GATAATACCAACTCGCTTATACGC |  |
| H_DLX2_RT_F | *DLX2*  NM_004405 | AGCCTGGACTTGGACACAGAGT | RT PCR |
| H_ DLX2_RT_R |  | GGGTTGCTGAGGTCACTGCTA |  |
| H_ DLX2_MSP_F |  | AATAAGAATAAATGTTAGATATTTCGA | MSP |
| H_ DLX2_MSP_R |  | ATAATTCGTACTTTCATATCCGAA |  |


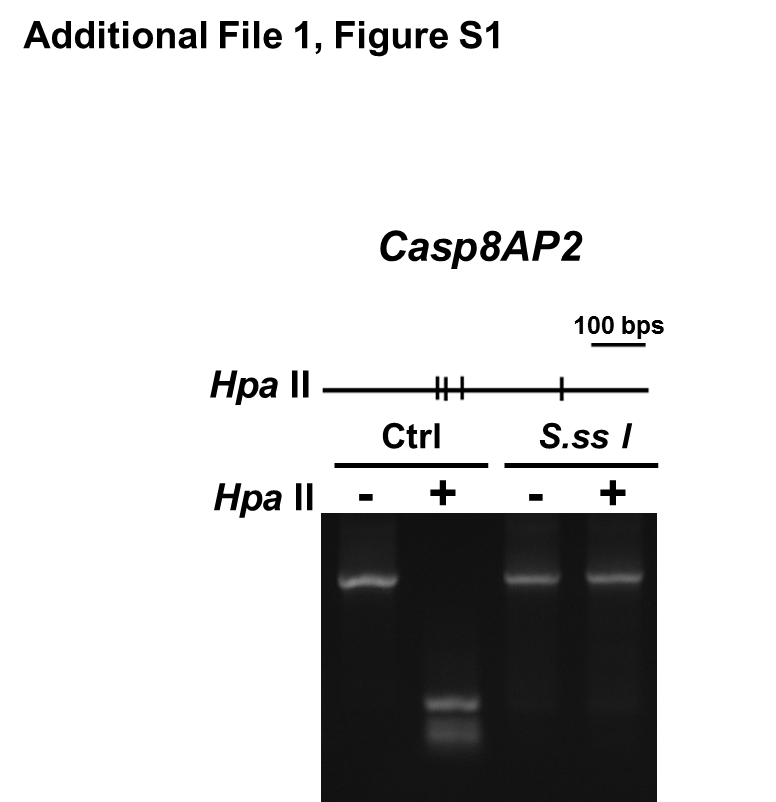


Figure S1. Restriction validation of *in vitro Casp8AP2* methylation. *In vitro* methylated *Casp8AP2* DNA (Treated with *S.ss*I methylase) and the untreated control (Ctrl) were subjected to restriction analysis by *Hpa*II enzyme. After restriction, the DNAs were gel-analyzed. The methylated DNA resisted to *Hpa*II restriction.


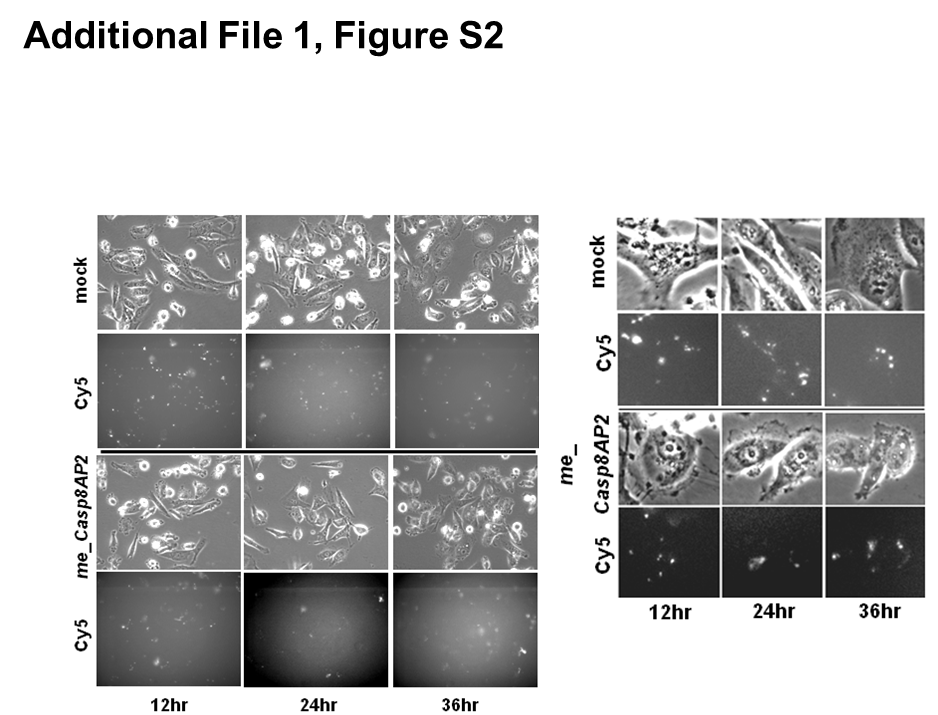


Figure S2. Transfection and localization of the transfected DNAs. Left Panel, Cells were transfected with Fluorescent Arrest-IN (FAI) transfection reagent (Thermo Scientific), which is conjugated with rhodamine, to determine the transfection efficiency. Right panel, Transfected DNAs were tracked with the *label*IT Tracker reagent, Intracellular Nucleic Acid Localization kit (Mirus), as described by the manufacturer.


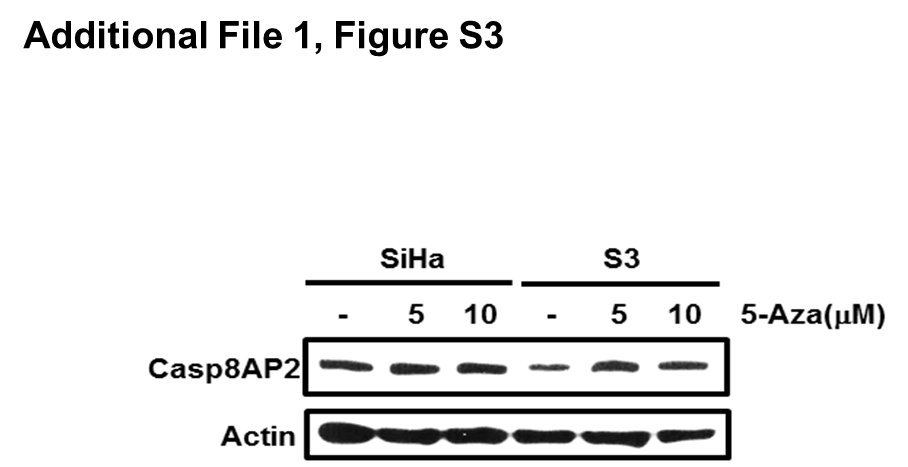


Figure S3. Restored Casp8AP2 expression after 5-Aza treatment. Control SiHa cells and its derived S3 cells were treated with designated concentration of 5-Aza, a demethylation agent. After 5 days and treated twice, proteins were isolated and analyzed by Western blotting.


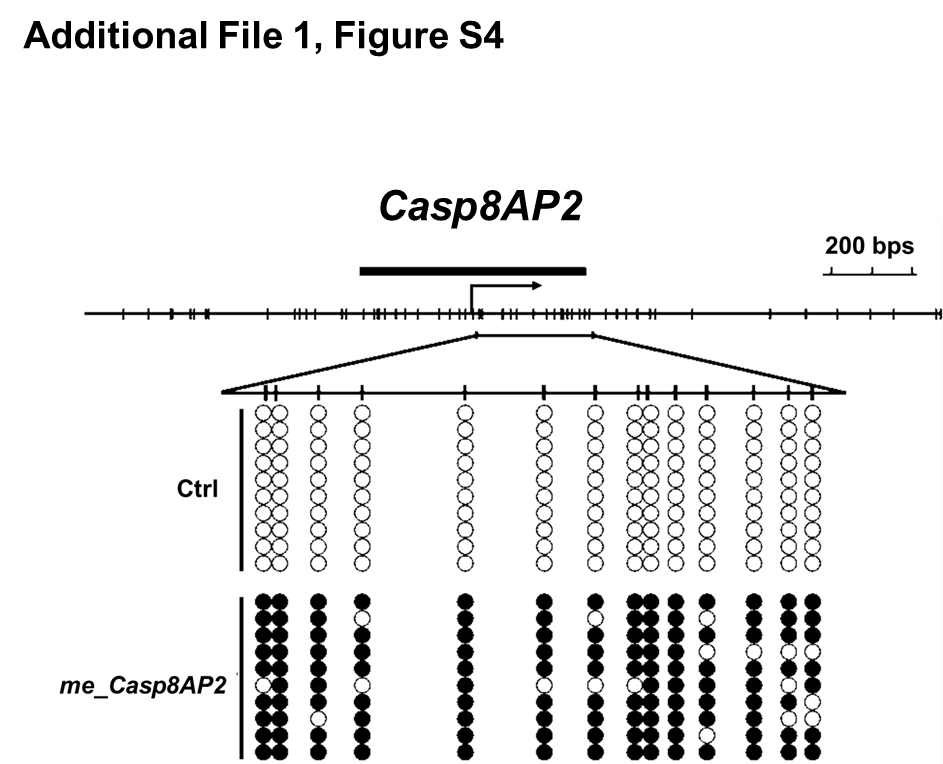


Figure S4. Bisulfite sequence analysis of the increased DNA methylation after targeted DNA methylation. After transfected with targeted methylated DNA (horizontal bar), genomic DNAs were collected from mock-treated control cells (Ctrl) or the methylated DNA treated (*me_Casp8AP2*) cells. The DNAs were then bisulfite-converted, POCR amplified and subcloned. Ten clones from each treatment were sequenced and the methylation states from CpG loci (vertical short bars) were depicted by circles. Open circles indicated the unmethylated ones and the filled ones represented the methylated loci.


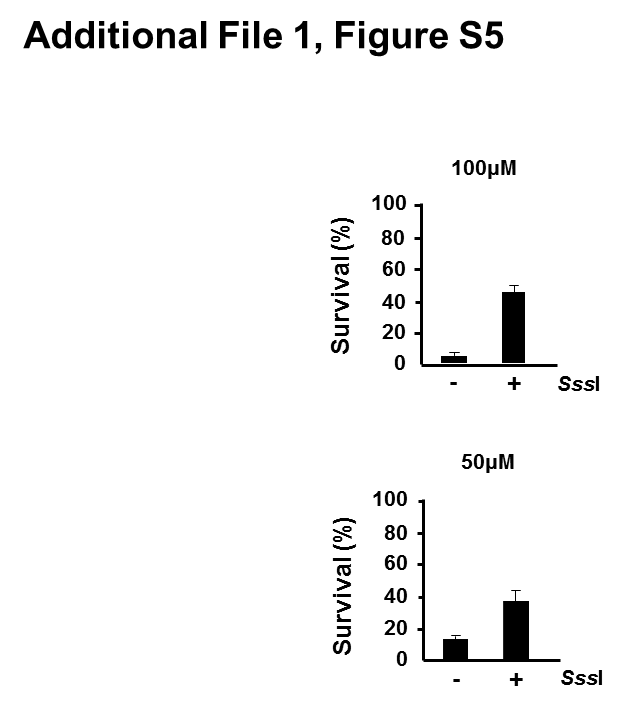


Figure S5. Increased MSC survival after targeted *Casp8AP2* methylation. MSCs were transfected with *in vitro* methylated (*S.ss*I) or unmethylated *Casp8AP2* DNA and then challenged with designated concentration of cisplatin (upper panel) and taxol (lower panel). Cell survival after drug treatment was detected by MTT assay.
